# *rac*-6-Hy­droxy-4-(4-nitro­phen­yl)-5-(2-thienyl­carbon­yl)-6-(trifluoro­meth­yl)-3,4,5,6-tetra­hydro­pyrimidin-2(1*H*)-one monohydrate

**DOI:** 10.1107/S1600536810041589

**Published:** 2010-10-23

**Authors:** Jian-Li Zhang, Hong-Sheng Wang, Yu-Jiao Niu, Feng-Xiang Zhu

**Affiliations:** a College of Chemistry and Chemical Engineering, Xuchang University, Xuchang, Henan Province, 461000, People’s Republic of China

## Abstract

The title compound, C_16_H_12_F_3_N_3_O_5_S·H_2_O, was prepared by reaction of 4-nitro­benzaldehyde, 4,4,4-trifluoro-1-(thio­phen-2-yl)butane-1,3-dione and urea. The asymmetric unit contains two independent mol­ecules, with essentially identical geom­etries and conformations. The dihydro­pyrimidine rings adopt a half-chair conformation. The dihedral angles between the benzene ring and the thio­phene ring are 54.82 (8) and 58.72 (8)° in the two mol­ecules. The mol­ecular conformation of one of the mol­ecules is stabilized by two intra­molecular O—H⋯O hydrogen bonds, generating an *S*(6) ring. The crystal structure is stabilized by inter­molecular O—H⋯O and N—H⋯O hydrogen bonds.

## Related literature

For the bioactivity of dihydro­pyrimidines, see: Brier *et al.* (2004 ▶); Cochran *et al.* (2005 ▶); Moran *et al.* (2007 ▶); Zorkun *et al.* (2006 ▶). For the bioactivity of organofluorine compounds, see: Hermann *et al.* (2003 ▶); Ulrich (2004 ▶).
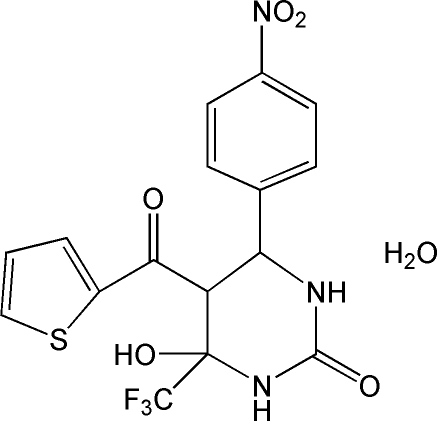


## Experimental

### 

#### Crystal data

C_16_H_12_F_3_N_3_O_5_S·H_2_O
*M* *_r_* = 433.36
Orthorhombic, 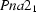

*a* = 14.1640 (13) Å
*b* = 9.136 (1) Å
*c* = 27.459 (3) Å
*V* = 3553.3 (6) Å^3^
*Z* = 8
Mo *K*α radiation
μ = 0.26 mm^−1^
*T* = 113 K
0.24 × 0.20 × 0.18 mm

#### Data collection

Rigaku Saturn724 CCD diffractometer
Absorption correction: multi-scan (*CrystalClear-SM Expert*; Rigaku, 2009 ▶) *T*_min_ = 0.941, *T*_max_ = 0.956
34133 measured reflections
7861 independent reflections
7466 reflections with *I* > 2σ(*I*)
*R*_int_ = 0.031

#### Refinement

*R*[*F* ^2^ > 2σ(*F* ^2^)] = 0.037
*wR*(*F* ^2^) = 0.091
*S* = 1.05
7861 reflections
563 parameters
1 restraint
H atoms treated by a mixture of independent and constrained refinement
Δρ_max_ = 0.37 e Å^−3^
Δρ_min_ = −0.27 e Å^−3^
Absolute structure: Flack (1983 ▶), 3539 Friedel pairs
Flack parameter: −0.01 (5)

### 

Data collection: *CrystalClear-SM Expert* (Rigaku, 2009 ▶); cell refinement: *CrystalClear-SM Expert*; data reduction: *CrystalClear-SM Expert*; program(s) used to solve structure: *SHELXS97* (Sheldrick, 2008 ▶); program(s) used to refine structure: *SHELXL97* (Sheldrick, 2008 ▶); molecular graphics: *CrystalStructure* (Rigaku, 2009 ▶); software used to prepare material for publication: *CrystalStructure*.

## Supplementary Material

Crystal structure: contains datablocks global, I. DOI: 10.1107/S1600536810041589/fj2348sup1.cif

Structure factors: contains datablocks I. DOI: 10.1107/S1600536810041589/fj2348Isup2.hkl

Additional supplementary materials:  crystallographic information; 3D view; checkCIF report

## Figures and Tables

**Table 1:** Hydrogen-bond geometry (Å, °)

*D*—H⋯*A*	*D*—H	H⋯*A*	*D*⋯*A*	*D*—H⋯*A*
O12—H12*A*⋯O6	0.85 (3)	2.28 (3)	2.867 (2)	126 (2)
O12—H12*A*⋯O8	0.85 (3)	2.20 (3)	2.949 (2)	147 (3)
O12—H12*B*⋯O7^i^	0.79 (3)	2.11 (3)	2.852 (2)	156 (3)
N1—H1⋯O12^ii^	0.99 (2)	2.00 (2)	2.969 (2)	168.5 (19)
N2—H2⋯O12^iii^	0.80 (3)	2.15 (3)	2.905 (2)	159 (2)
N5—H5⋯O11	0.82 (3)	2.15 (3)	2.903 (2)	153 (3)
N4—H4⋯O11^iv^	0.84 (3)	2.17 (3)	2.991 (2)	166 (2)
O6—H6⋯O2^v^	0.82 (3)	1.86 (3)	2.684 (2)	179 (3)
O1—H1*A*⋯O7^vi^	0.93 (4)	1.76 (4)	2.684 (2)	175 (4)
O11—H11*A*⋯O2^vii^	0.81 (3)	2.06 (3)	2.849 (2)	166 (3)
O11—H11*B*⋯O3^viii^	0.83 (3)	2.23 (3)	2.968 (2)	148 (3)
O11—H11*B*⋯O1^viii^	0.83 (3)	2.25 (3)	2.863 (2)	131 (3)

Symmetry codes: (i) 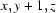; (ii) 
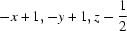
; (iii) 
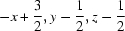
; (iv) 
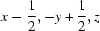
; (v) 
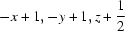
; (vi) 
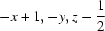
; (vii) 
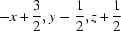
; (viii) 
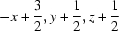
.

**Fig. 1.**
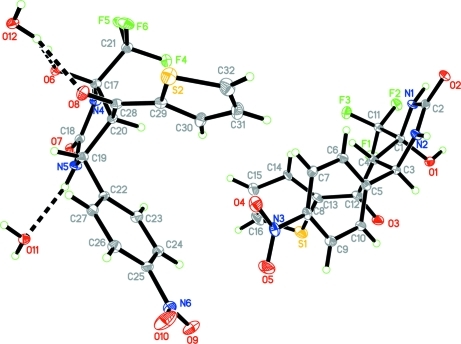
Molecular configuration and atom numbering scheme for (I), with displacement ellipsoids drawn at the 30% probability level.

**Fig. 2.**
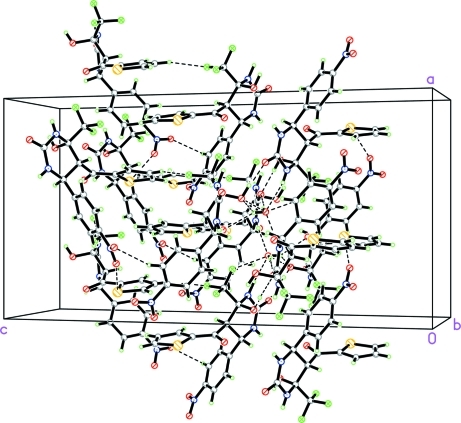
The packing diagram of the title compound. Intermolecular hydrogen bonds are shown as dashed line.

## supplementary crystallographic information

### Comment

Dihydropyrimidine (DHPM) derivatives can be used as potential calcium channel blockers (Zorkun *et al.*, 2006), inhibitors of mitotic kinesin Eg5 for treating cancer (Cochran *et al.*, 2005; Brier *et al.*, 2004) and as TRPA1 modulators for treating pain (Moran *et al.*, 2007). In addition, compounds that contain fluorine have special bioactivity, *e.g.* flumioxazin is a widely used herbicide (Hermann *et al.*, 2003; Ulrich, 2004). This led us to focus our attention on the synthesis and bioactivity of these important fused perfluoroalkylated heterocyclic compounds. During the synthesis of DHPM derivatives, the title compound, an intermediate C_16_H_14_F_3_N_3_O_6_S (I) was isolated and the structure confirmed by X-ray diffraction, in order to elucidate the reaction mechanism.

The title compound crystallizes with two independent molecules in the asymmetric unit. In the structure of the title molecule, the dihydropyrimidine ring adopts a half-chair conformation. The molecular conformation of one of the molecules is stabilized by two intramolecular O—H···O hydrogen bond, generating an S(6) ring. The crystal structure is stabilized by nine intermolecular hydrogen bonds (six O—H···O and three N—H···O) (Table 1). The dihedral angles between the pyridine ring and the thiophene ring are 54.82 (8)° and 58.72 (8)° in the two molecules.

### Experimental

The title compound was synthesized refluxing for 3 h a stirred solution of 4-nitrobenzaldehyde (0.30 g, 2 mmol), 4,4,4-trifluoro-1- (thiophen-2-yl)butane-1,3-dione (0.51 g, 2.3 mmol) and urea (0.18 g, 3 mmol) in 3 ml of anhydrous ethanol, the reaction catalyzed by sulfamic acid (0.06 g). The solvent was evaporated *in vacuo* and the residue was washed with water. The title compound was recrystallized from 50% aqueous ethanol and single crystals of (I) were obtained by slow evaporation.

### Refinement

Hydrogen atoms involved in hydrogen-bonding inetractions were located by difference methods and their positional and isotropic displacement parameters were refined. Other H atoms were placed in calculated positions, with C—H(aromatic) = 0.95 Å and C—H(aliphatic) = 1.00 Å, and treated as riding, with *U*_iso_(H) = 1.2*U*eq(C).

### Crystal data

**Table:**

C_16_H_12_F_3_N_3_O_5_S·H_2_O	*F*(000) = 1776
*M**_r_* = 433.36	*D*_x_ = 1.620 Mg m^−^^3^
Orthorhombic, *P**n**a*2_1_	Mo *K*α radiation, λ = 0.71075 Å
Hall symbol: P 2c -2n	Cell parameters from 9674 reflections
*a* = 14.1640 (13) Å	θ = 1.6–27.9°
*b* = 9.136 (1) Å	µ = 0.26 mm^−^^1^
*c* = 27.459 (3) Å	*T* = 113 K
*V* = 3553.3 (6) Å^3^	Prism, colorless
*Z* = 8	0.24 × 0.20 × 0.18 mm

### Data collection

**Table:**

Rigaku Saturn724 CCD diffractometer	7861 independent reflections
Radiation source: rotating anode	7466 reflections with *I* > 2σ(*I*)
multilayer	*R*_int_ = 0.031
Detector resolution: 14.222 pixels mm^-1^	θ_max_ = 27.9°, θ_min_ = 1.5°
ω scans	*h* = −18→18
Absorption correction: multi-scan (*CrystalClear-SM Expert*; Rigaku, 2009)	*k* = −12→11
*T*_min_ = 0.941, *T*_max_ = 0.956	*l* = −35→36
34133 measured reflections	

### Refinement

**Table:**

Refinement on *F*^2^	Secondary atom site location: difference Fourier map
Least-squares matrix: full	Hydrogen site location: inferred from neighbouring sites
*R*[*F*^2^ > 2σ(*F*^2^)] = 0.037	H atoms treated by a mixture of independent and constrained refinement
*wR*(*F*^2^) = 0.091	*w* = 1/[σ^2^(*F*_o_^2^) + (0.0567*P*)^2^] where *P* = (*F*_o_^2^ + 2*F*_c_^2^)/3
*S* = 1.05	(Δ/σ)_max_ < 0.001
7861 reflections	Δρ_max_ = 0.37 e Å^−^^3^
563 parameters	Δρ_min_ = −0.27 e Å^−^^3^
1 restraint	Absolute structure: Flack (1983), 3539 Friedel pairs
Primary atom site location: structure-invariant direct methods	Flack parameter: −0.01 (5)

### Special details

**Table:**

Geometry. All e.s.d.'s (except the e.s.d. in the dihedral angle between two l.s. planes) are estimated using the full covariance matrix. The cell e.s.d.'s are taken into account individually in the estimation of e.s.d.'s in distances, angles and torsion angles; correlations between e.s.d.'s in cell parameters are only used when they are defined by crystal symmetry. An approximate (isotropic) treatment of cell e.s.d.'s is used for estimating e.s.d.'s involving l.s. planes.
Refinement. Refinement of *F*^2^ against ALL reflections. The weighted *R*-factor *wR* and goodness of fit *S* are based on *F*^2^, conventional *R*-factors *R* are based on *F*, with *F* set to zero for negative *F*^2^. The threshold expression of *F*^2^ > σ(*F*^2^) is used only for calculating *R*-factors(gt) *etc*. and is not relevant to the choice of reflections for refinement. *R*-factors based on *F*^2^ are statistically about twice as large as those based on *F*, and *R*- factors based on ALL data will be even larger.

### Fractional atomic coordinates and isotropic or equivalent isotropic displacement parameters (Å^2^)

**Table:**

	*x*	*y*	*z*	*U*_iso_*/*U*_eq_	
S1	0.60772 (5)	−0.35697 (6)	0.08791 (3)	0.03689 (15)	
F1	0.37968 (10)	−0.23469 (13)	0.00751 (6)	0.0331 (3)	
F2	0.27429 (8)	−0.07998 (14)	−0.01598 (5)	0.0274 (3)	
F3	0.35284 (9)	−0.04033 (14)	0.04972 (5)	0.0292 (3)	
O1	0.45567 (10)	−0.08713 (15)	−0.06818 (5)	0.0212 (3)	
O2	0.43350 (10)	0.36232 (14)	−0.06593 (5)	0.0211 (3)	
O3	0.59480 (11)	−0.23377 (16)	−0.01125 (6)	0.0282 (3)	
O4	0.89623 (12)	0.33821 (17)	0.13355 (6)	0.0335 (4)	
O5	0.96906 (14)	0.1399 (2)	0.11251 (7)	0.0467 (5)	
N1	0.40188 (13)	0.13684 (17)	−0.03407 (6)	0.0180 (3)	
N2	0.55597 (13)	0.2246 (2)	−0.04098 (7)	0.0203 (4)	
N3	0.90201 (14)	0.22519 (19)	0.10965 (7)	0.0249 (4)	
C1	0.43619 (13)	−0.01001 (19)	−0.02507 (7)	0.0165 (4)	
C2	0.46358 (14)	0.2445 (2)	−0.04839 (7)	0.0183 (4)	
C3	0.60082 (14)	0.0912 (2)	−0.02294 (7)	0.0173 (4)	
H3	0.6249	0.0323	−0.0510	0.021*	
C4	0.52713 (14)	0.00176 (19)	0.00539 (7)	0.0172 (4)	
H4A	0.5117	0.0564	0.0359	0.021*	
C5	0.68228 (15)	0.1286 (2)	0.01111 (7)	0.0182 (4)	
C6	0.67296 (15)	0.2434 (2)	0.04432 (8)	0.0216 (4)	
H6A	0.6169	0.3005	0.0445	0.026*	
C7	0.74425 (16)	0.2748 (2)	0.07696 (7)	0.0214 (4)	
H7	0.7383	0.3535	0.0994	0.026*	
C8	0.82467 (15)	0.1886 (2)	0.07617 (7)	0.0205 (4)	
C9	0.83640 (15)	0.0731 (2)	0.04422 (8)	0.0226 (4)	
H9	0.8922	0.0154	0.0447	0.027*	
C10	0.76357 (14)	0.0438 (2)	0.01115 (7)	0.0202 (4)	
H10	0.7698	−0.0346	−0.0114	0.024*	
C11	0.35973 (15)	−0.0915 (2)	0.00413 (7)	0.0203 (4)	
C12	0.56951 (15)	−0.1468 (2)	0.01993 (8)	0.0206 (4)	
C13	0.57782 (15)	−0.1808 (2)	0.07158 (8)	0.0217 (4)	
C14	0.56852 (15)	−0.0877 (2)	0.11324 (7)	0.0241 (4)	
H14	0.5543	0.0139	0.1124	0.029*	
C15	0.58417 (19)	−0.1726 (3)	0.15664 (9)	0.0361 (6)	
H15	0.5803	−0.1326	0.1885	0.043*	
C16	0.60494 (19)	−0.3148 (3)	0.14778 (10)	0.0407 (7)	
H16	0.6168	−0.3842	0.1728	0.049*	
S2	0.87505 (5)	0.87073 (6)	0.23286 (2)	0.03609 (15)	
F4	0.62278 (9)	0.54205 (14)	0.26486 (4)	0.0278 (3)	
F5	0.64890 (10)	0.73570 (13)	0.30749 (6)	0.0319 (3)	
F6	0.54339 (9)	0.57986 (14)	0.33042 (5)	0.0261 (3)	
O6	0.72494 (11)	0.58706 (15)	0.38319 (5)	0.0206 (3)	
O7	0.70213 (11)	0.13746 (14)	0.38037 (5)	0.0213 (3)	
O8	0.86907 (11)	0.72990 (16)	0.32953 (6)	0.0253 (3)	
O9	1.16517 (13)	0.15713 (17)	0.18214 (6)	0.0363 (4)	
O10	1.23927 (14)	0.3536 (2)	0.20196 (8)	0.0558 (6)	
N4	0.67149 (13)	0.36304 (16)	0.34850 (6)	0.0174 (3)	
N5	0.82537 (13)	0.27411 (19)	0.35508 (7)	0.0201 (4)	
N6	1.17126 (14)	0.2717 (2)	0.20498 (7)	0.0264 (4)	
C17	0.70562 (14)	0.5113 (2)	0.34018 (7)	0.0181 (4)	
C18	0.73277 (14)	0.2537 (2)	0.36261 (7)	0.0172 (4)	
C19	0.87044 (14)	0.4083 (2)	0.33745 (7)	0.0194 (4)	
H19	0.8951	0.4658	0.3657	0.023*	
C20	0.79665 (14)	0.49985 (19)	0.30975 (7)	0.0173 (4)	
H20	0.7814	0.4485	0.2786	0.021*	
C21	0.62941 (15)	0.5919 (2)	0.31056 (8)	0.0208 (4)	
C22	0.95101 (14)	0.37086 (19)	0.30321 (7)	0.0173 (4)	
C23	0.94011 (15)	0.2601 (2)	0.26815 (8)	0.0222 (4)	
H23	0.8829	0.2060	0.2669	0.027*	
C24	1.01085 (15)	0.2290 (2)	0.23565 (7)	0.0218 (4)	
H24	1.0034	0.1537	0.2121	0.026*	
C25	1.09372 (15)	0.3103 (2)	0.23800 (7)	0.0205 (4)	
C26	1.10671 (16)	0.4235 (2)	0.27159 (8)	0.0236 (4)	
H26	1.1635	0.4789	0.2720	0.028*	
C27	1.03457 (14)	0.4527 (2)	0.30426 (8)	0.0211 (4)	
H27	1.0419	0.5287	0.3275	0.025*	
C28	0.83971 (14)	0.6501 (2)	0.29737 (7)	0.0190 (4)	
C29	0.84472 (15)	0.6933 (2)	0.24656 (8)	0.0210 (4)	
C30	0.82926 (16)	0.6111 (2)	0.20372 (8)	0.0251 (4)	
H30	0.8130	0.5102	0.2032	0.030*	
C31	0.84114 (19)	0.6988 (3)	0.16187 (9)	0.0356 (6)	
H31	0.8340	0.6624	0.1297	0.043*	
C32	0.8636 (2)	0.8396 (3)	0.17194 (10)	0.0391 (6)	
H32	0.8720	0.9128	0.1478	0.047*	
O11	0.98547 (11)	0.12871 (16)	0.40001 (6)	0.0222 (3)	
O12	0.78322 (11)	0.87168 (16)	0.41523 (6)	0.0219 (3)	
H12A	0.785 (2)	0.818 (3)	0.3900 (11)	0.043 (8)*	
H12B	0.777 (2)	0.950 (4)	0.4040 (12)	0.058 (10)*	
H1	0.3382 (17)	0.146 (2)	−0.0483 (9)	0.020 (6)*	
H2	0.5907 (17)	0.283 (3)	−0.0528 (9)	0.022 (6)*	
H5	0.859 (2)	0.209 (3)	0.3656 (10)	0.035 (7)*	
H4	0.615 (2)	0.362 (3)	0.3582 (9)	0.028 (7)*	
H6	0.6766 (19)	0.603 (3)	0.3989 (10)	0.029 (7)*	
H1A	0.399 (3)	−0.106 (4)	−0.0845 (14)	0.073 (11)*	
H11A	0.9996 (19)	0.051 (3)	0.4119 (10)	0.034 (8)*	
H11B	0.982 (2)	0.188 (3)	0.4227 (11)	0.042 (8)*	

### Atomic displacement parameters (Å^2^)

**Table:**

	*U*^11^	*U*^22^	*U*^33^	*U*^12^	*U*^13^	*U*^23^
S1	0.0438 (4)	0.0252 (3)	0.0416 (4)	0.0070 (2)	−0.0030 (3)	0.0096 (2)
F1	0.0316 (8)	0.0147 (6)	0.0531 (9)	−0.0018 (5)	0.0029 (7)	0.0088 (6)
F2	0.0176 (6)	0.0335 (7)	0.0311 (7)	−0.0052 (5)	−0.0027 (5)	0.0088 (5)
F3	0.0302 (7)	0.0358 (7)	0.0216 (6)	−0.0038 (5)	0.0023 (6)	0.0011 (5)
O1	0.0194 (8)	0.0232 (7)	0.0210 (7)	0.0013 (6)	−0.0034 (6)	−0.0063 (6)
O2	0.0199 (8)	0.0173 (6)	0.0261 (7)	−0.0003 (5)	−0.0042 (6)	0.0053 (5)
O3	0.0308 (9)	0.0224 (7)	0.0313 (8)	0.0083 (6)	−0.0068 (7)	−0.0071 (6)
O4	0.0384 (10)	0.0298 (8)	0.0322 (9)	−0.0065 (7)	−0.0105 (8)	−0.0042 (7)
O5	0.0321 (11)	0.0551 (11)	0.0528 (12)	0.0129 (8)	−0.0214 (9)	−0.0176 (10)
N1	0.0175 (9)	0.0135 (8)	0.0230 (8)	0.0008 (6)	−0.0030 (7)	0.0024 (6)
N2	0.0158 (9)	0.0194 (8)	0.0256 (9)	−0.0023 (7)	−0.0031 (7)	0.0076 (7)
N3	0.0253 (10)	0.0276 (9)	0.0219 (9)	−0.0060 (7)	−0.0058 (8)	0.0032 (7)
C1	0.0171 (10)	0.0132 (8)	0.0192 (9)	−0.0001 (7)	−0.0034 (8)	−0.0014 (7)
C2	0.0183 (10)	0.0200 (9)	0.0167 (9)	−0.0010 (8)	0.0011 (8)	0.0017 (7)
C3	0.0160 (10)	0.0163 (8)	0.0196 (9)	0.0014 (7)	−0.0007 (8)	0.0005 (7)
C4	0.0180 (10)	0.0138 (8)	0.0199 (9)	0.0017 (7)	−0.0020 (8)	−0.0001 (7)
C5	0.0181 (10)	0.0180 (9)	0.0185 (9)	−0.0018 (7)	−0.0011 (8)	0.0031 (7)
C6	0.0189 (11)	0.0213 (9)	0.0246 (10)	0.0016 (8)	−0.0020 (8)	−0.0015 (8)
C7	0.0236 (11)	0.0203 (9)	0.0204 (10)	−0.0017 (8)	−0.0001 (8)	−0.0035 (8)
C8	0.0198 (11)	0.0237 (9)	0.0180 (9)	−0.0054 (8)	−0.0038 (8)	0.0039 (7)
C9	0.0201 (11)	0.0214 (9)	0.0265 (10)	0.0001 (8)	−0.0010 (8)	−0.0006 (8)
C10	0.0199 (11)	0.0184 (9)	0.0222 (10)	0.0010 (8)	−0.0027 (8)	−0.0009 (8)
C11	0.0209 (11)	0.0185 (9)	0.0216 (10)	0.0010 (7)	−0.0022 (8)	0.0017 (7)
C12	0.0154 (10)	0.0169 (9)	0.0296 (11)	−0.0006 (7)	−0.0048 (9)	0.0002 (8)
C13	0.0184 (11)	0.0163 (9)	0.0303 (10)	−0.0008 (8)	−0.0060 (9)	0.0055 (7)
C14	0.0208 (11)	0.0311 (10)	0.0205 (10)	0.0064 (8)	−0.0012 (8)	0.0112 (8)
C15	0.0361 (15)	0.0453 (14)	0.0269 (12)	0.0058 (11)	−0.0007 (11)	0.0050 (10)
C16	0.0357 (15)	0.0459 (16)	0.0405 (15)	0.0049 (12)	−0.0027 (12)	0.0243 (12)
S2	0.0525 (4)	0.0218 (2)	0.0340 (3)	−0.0066 (2)	0.0044 (3)	0.0062 (2)
F4	0.0294 (7)	0.0327 (7)	0.0213 (6)	0.0030 (5)	−0.0034 (5)	0.0000 (5)
F5	0.0323 (7)	0.0156 (5)	0.0478 (8)	0.0023 (5)	−0.0039 (7)	0.0052 (6)
F6	0.0173 (6)	0.0315 (6)	0.0296 (6)	0.0051 (5)	0.0031 (5)	0.0058 (5)
O6	0.0196 (8)	0.0229 (7)	0.0193 (7)	−0.0004 (6)	0.0025 (6)	−0.0042 (6)
O7	0.0226 (8)	0.0160 (7)	0.0253 (7)	0.0012 (5)	0.0047 (6)	0.0047 (5)
O8	0.0287 (9)	0.0236 (7)	0.0236 (7)	−0.0073 (6)	0.0028 (6)	−0.0041 (6)
O9	0.0433 (11)	0.0295 (8)	0.0361 (9)	0.0045 (7)	0.0192 (8)	−0.0046 (7)
O10	0.0339 (11)	0.0690 (14)	0.0646 (14)	−0.0199 (10)	0.0273 (10)	−0.0271 (11)
N4	0.0145 (9)	0.0147 (7)	0.0231 (9)	0.0011 (6)	0.0033 (7)	0.0030 (6)
N5	0.0184 (9)	0.0171 (8)	0.0247 (9)	0.0038 (7)	0.0033 (7)	0.0054 (7)
N6	0.0239 (10)	0.0317 (9)	0.0238 (9)	0.0025 (8)	0.0045 (8)	0.0008 (8)
C17	0.0184 (11)	0.0150 (8)	0.0210 (10)	−0.0004 (7)	0.0017 (8)	−0.0002 (7)
C18	0.0185 (10)	0.0163 (8)	0.0167 (9)	0.0006 (8)	0.0032 (8)	0.0000 (7)
C19	0.0202 (11)	0.0184 (9)	0.0196 (9)	0.0012 (8)	0.0037 (8)	0.0016 (7)
C20	0.0181 (10)	0.0153 (8)	0.0185 (9)	0.0002 (7)	0.0015 (8)	−0.0015 (7)
C21	0.0232 (11)	0.0168 (9)	0.0225 (10)	0.0002 (7)	0.0017 (8)	0.0005 (7)
C22	0.0179 (10)	0.0165 (9)	0.0174 (9)	0.0027 (7)	0.0032 (8)	0.0014 (7)
C23	0.0201 (11)	0.0199 (9)	0.0266 (11)	−0.0033 (8)	0.0001 (9)	−0.0031 (8)
C24	0.0222 (11)	0.0210 (9)	0.0222 (10)	0.0015 (8)	−0.0004 (9)	−0.0023 (8)
C25	0.0198 (11)	0.0215 (9)	0.0203 (9)	0.0048 (8)	0.0043 (8)	0.0029 (8)
C26	0.0185 (11)	0.0259 (10)	0.0264 (11)	−0.0031 (8)	0.0033 (9)	0.0021 (8)
C27	0.0220 (11)	0.0198 (9)	0.0215 (9)	−0.0002 (8)	−0.0009 (9)	−0.0017 (8)
C28	0.0177 (10)	0.0167 (9)	0.0226 (10)	0.0001 (7)	0.0031 (8)	0.0005 (7)
C29	0.0180 (11)	0.0192 (9)	0.0258 (10)	−0.0012 (8)	0.0022 (8)	0.0009 (8)
C30	0.0264 (12)	0.0282 (10)	0.0208 (10)	−0.0069 (8)	0.0033 (9)	−0.0006 (8)
C31	0.0333 (15)	0.0496 (15)	0.0238 (11)	−0.0044 (11)	0.0023 (10)	0.0034 (10)
C32	0.0449 (16)	0.0382 (13)	0.0342 (13)	−0.0031 (11)	0.0040 (12)	0.0150 (11)
O11	0.0262 (9)	0.0159 (7)	0.0245 (8)	0.0022 (6)	0.0001 (6)	−0.0019 (6)
O12	0.0239 (8)	0.0178 (7)	0.0242 (8)	0.0026 (6)	0.0004 (6)	−0.0026 (6)

### Geometric parameters (Å, °)

**Table:**

S1—C16	1.689 (3)	F4—C21	1.338 (2)
S1—C13	1.724 (2)	F5—C21	1.345 (2)
F1—C11	1.341 (2)	F6—C21	1.339 (2)
F2—C11	1.334 (2)	O6—C17	1.396 (2)
F3—C11	1.340 (2)	O6—H6	0.82 (3)
O1—C1	1.405 (2)	O7—C18	1.247 (2)
O1—H1A	0.93 (4)	O8—C28	1.218 (2)
O2—C2	1.254 (2)	O9—N6	1.224 (2)
O3—C12	1.222 (3)	O10—N6	1.222 (3)
O4—N3	1.226 (2)	N4—C18	1.379 (2)
O5—N3	1.231 (3)	N4—C17	1.456 (2)
N1—C2	1.373 (2)	N4—H4	0.84 (3)
N1—C1	1.448 (2)	N5—C18	1.341 (3)
N1—H1	0.99 (2)	N5—C19	1.465 (2)
N2—C2	1.337 (3)	N5—H5	0.82 (3)
N2—C3	1.462 (2)	N6—C25	1.467 (3)
N2—H2	0.80 (3)	C17—C21	1.539 (3)
N3—C8	1.469 (3)	C17—C20	1.540 (3)
C1—C4	1.540 (3)	C19—C22	1.518 (3)
C1—C11	1.540 (3)	C19—C20	1.539 (3)
C3—C5	1.524 (3)	C19—H19	1.0000
C3—C4	1.537 (3)	C20—C28	1.540 (3)
C3—H3	1.0000	C20—H20	1.0000
C4—C12	1.537 (3)	C22—C27	1.400 (3)
C4—H4A	1.0000	C22—C23	1.405 (3)
C5—C10	1.388 (3)	C23—C24	1.372 (3)
C5—C6	1.396 (3)	C23—H23	0.9500
C6—C7	1.380 (3)	C24—C25	1.391 (3)
C6—H6A	0.9500	C24—H24	0.9500
C7—C8	1.385 (3)	C25—C26	1.398 (3)
C7—H7	0.9500	C26—C27	1.386 (3)
C8—C9	1.382 (3)	C26—H26	0.9500
C9—C10	1.400 (3)	C27—H27	0.9500
C9—H9	0.9500	C28—C29	1.452 (3)
C10—H10	0.9500	C29—C30	1.413 (3)
C12—C13	1.457 (3)	C30—C31	1.411 (3)
C13—C14	1.432 (3)	C30—H30	0.9500
C14—C15	1.439 (3)	C31—C32	1.354 (4)
C14—H14	0.9500	C31—H31	0.9500
C15—C16	1.355 (4)	C32—H32	0.9500
C15—H15	0.9500	O11—H11A	0.81 (3)
C16—H16	0.9500	O11—H11B	0.83 (3)
S2—C32	1.705 (3)	O12—H12A	0.85 (3)
S2—C29	1.719 (2)	O12—H12B	0.79 (3)
			
C16—S1—C13	91.98 (12)	C17—O6—H6	111.8 (18)
C1—O1—H1A	109 (2)	C18—N4—C17	120.59 (17)
C2—N1—C1	119.93 (17)	C18—N4—H4	120.1 (17)
C2—N1—H1	114.3 (13)	C17—N4—H4	111.9 (16)
C1—N1—H1	116.7 (13)	C18—N5—C19	126.40 (17)
C2—N2—C3	126.19 (17)	C18—N5—H5	115 (2)
C2—N2—H2	116.9 (18)	C19—N5—H5	118 (2)
C3—N2—H2	115.4 (18)	O10—N6—O9	122.99 (19)
O4—N3—O5	123.40 (19)	O10—N6—C25	118.99 (18)
O4—N3—C8	118.49 (19)	O9—N6—C25	118.01 (18)
O5—N3—C8	118.10 (18)	O6—C17—N4	113.17 (16)
O1—C1—N1	112.75 (16)	O6—C17—C21	110.32 (15)
O1—C1—C4	109.18 (15)	N4—C17—C21	107.17 (16)
N1—C1—C4	107.98 (14)	O6—C17—C20	109.18 (16)
O1—C1—C11	109.54 (15)	N4—C17—C20	107.44 (15)
N1—C1—C11	107.52 (16)	C21—C17—C20	109.46 (16)
C4—C1—C11	109.84 (16)	O7—C18—N5	121.27 (18)
O2—C2—N2	120.51 (18)	O7—C18—N4	120.53 (18)
O2—C2—N1	120.57 (18)	N5—C18—N4	118.16 (17)
N2—C2—N1	118.82 (18)	N5—C19—C22	110.10 (16)
N2—C3—C5	110.45 (16)	N5—C19—C20	108.79 (16)
N2—C3—C4	108.65 (16)	C22—C19—C20	109.09 (16)
C5—C3—C4	108.86 (16)	N5—C19—H19	109.6
N2—C3—H3	109.6	C22—C19—H19	109.6
C5—C3—H3	109.6	C20—C19—H19	109.6
C4—C3—H3	109.6	C19—C20—C28	108.92 (16)
C3—C4—C12	109.60 (16)	C19—C20—C17	109.72 (15)
C3—C4—C1	109.30 (15)	C28—C20—C17	113.00 (15)
C12—C4—C1	113.97 (15)	C19—C20—H20	108.4
C3—C4—H4A	107.9	C28—C20—H20	108.4
C12—C4—H4A	107.9	C17—C20—H20	108.4
C1—C4—H4A	107.9	F4—C21—F6	106.85 (17)
C10—C5—C6	119.84 (19)	F4—C21—F5	106.75 (16)
C10—C5—C3	120.19 (17)	F6—C21—F5	107.02 (16)
C6—C5—C3	119.87 (18)	F4—C21—C17	112.44 (16)
C7—C6—C5	120.74 (19)	F6—C21—C17	112.55 (16)
C7—C6—H6A	119.6	F5—C21—C17	110.88 (16)
C5—C6—H6A	119.6	C27—C22—C23	119.43 (18)
C6—C7—C8	118.24 (18)	C27—C22—C19	120.17 (17)
C6—C7—H7	120.9	C23—C22—C19	120.28 (18)
C8—C7—H7	120.9	C24—C23—C22	120.99 (19)
C9—C8—C7	122.89 (19)	C24—C23—H23	119.5
C9—C8—N3	118.78 (19)	C22—C23—H23	119.5
C7—C8—N3	118.29 (18)	C23—C24—C25	118.40 (19)
C8—C9—C10	117.99 (19)	C23—C24—H24	120.8
C8—C9—H9	121.0	C25—C24—H24	120.8
C10—C9—H9	121.0	C24—C25—C26	122.47 (19)
C5—C10—C9	120.30 (18)	C24—C25—N6	118.35 (18)
C5—C10—H10	119.9	C26—C25—N6	119.15 (19)
C9—C10—H10	119.9	C27—C26—C25	118.2 (2)
F2—C11—F3	107.04 (16)	C27—C26—H26	120.9
F2—C11—F1	107.27 (16)	C25—C26—H26	120.9
F3—C11—F1	106.93 (16)	C26—C27—C22	120.49 (19)
F2—C11—C1	112.58 (16)	C26—C27—H27	119.8
F3—C11—C1	111.65 (16)	C22—C27—H27	119.8
F1—C11—C1	111.06 (16)	O8—C28—C29	121.14 (18)
O3—C12—C13	121.33 (19)	O8—C28—C20	120.58 (18)
O3—C12—C4	120.45 (19)	C29—C28—C20	118.28 (17)
C13—C12—C4	118.22 (18)	C30—C29—C28	130.41 (18)
C14—C13—C12	130.08 (18)	C30—C29—S2	110.97 (16)
C14—C13—S1	111.70 (15)	C28—C29—S2	118.61 (15)
C12—C13—S1	118.18 (16)	C31—C30—C29	110.97 (19)
C13—C14—C15	109.10 (19)	C31—C30—H30	124.5
C13—C14—H14	125.4	C29—C30—H30	124.5
C15—C14—H14	125.4	C32—C31—C30	113.7 (2)
C16—C15—C14	113.7 (2)	C32—C31—H31	123.2
C16—C15—H15	123.2	C30—C31—H31	123.2
C14—C15—H15	123.2	C31—C32—S2	112.42 (19)
C15—C16—S1	113.50 (19)	C31—C32—H32	123.8
C15—C16—H16	123.3	S2—C32—H32	123.8
S1—C16—H16	123.3	H11A—O11—H11B	107 (3)
C32—S2—C29	91.93 (12)	H12A—O12—H12B	102 (3)
			
C2—N1—C1—O1	−75.5 (2)	C18—N4—C17—O6	−75.2 (2)
C2—N1—C1—C4	45.2 (2)	C18—N4—C17—C21	162.97 (17)
C2—N1—C1—C11	163.65 (17)	C18—N4—C17—C20	45.4 (2)
C3—N2—C2—O2	−177.07 (18)	C19—N5—C18—O7	−175.88 (19)
C3—N2—C2—N1	6.4 (3)	C19—N5—C18—N4	6.5 (3)
C1—N1—C2—O2	165.12 (18)	C17—N4—C18—O7	163.48 (18)
C1—N1—C2—N2	−18.3 (3)	C17—N4—C18—N5	−18.9 (3)
C2—N2—C3—C5	−142.2 (2)	C18—N5—C19—C22	−142.0 (2)
C2—N2—C3—C4	−22.9 (3)	C18—N5—C19—C20	−22.5 (3)
N2—C3—C4—C12	173.84 (16)	N5—C19—C20—C28	172.20 (16)
C5—C3—C4—C12	−65.8 (2)	C22—C19—C20—C28	−67.68 (19)
N2—C3—C4—C1	48.3 (2)	N5—C19—C20—C17	48.0 (2)
C5—C3—C4—C1	168.61 (15)	C22—C19—C20—C17	168.15 (15)
O1—C1—C4—C3	63.20 (18)	O6—C17—C20—C19	63.79 (19)
N1—C1—C4—C3	−59.71 (19)	N4—C17—C20—C19	−59.30 (19)
C11—C1—C4—C3	−176.67 (15)	C21—C17—C20—C19	−175.35 (15)
O1—C1—C4—C12	−59.8 (2)	O6—C17—C20—C28	−58.0 (2)
N1—C1—C4—C12	177.29 (17)	N4—C17—C20—C28	178.94 (16)
C11—C1—C4—C12	60.3 (2)	C21—C17—C20—C28	62.9 (2)
N2—C3—C5—C10	−143.50 (19)	O6—C17—C21—F4	166.64 (15)
C4—C3—C5—C10	97.3 (2)	N4—C17—C21—F4	−69.8 (2)
N2—C3—C5—C6	40.2 (2)	C20—C17—C21—F4	46.5 (2)
C4—C3—C5—C6	−79.0 (2)	O6—C17—C21—F6	−72.6 (2)
C10—C5—C6—C7	0.8 (3)	N4—C17—C21—F6	51.0 (2)
C3—C5—C6—C7	177.05 (18)	C20—C17—C21—F6	167.20 (15)
C5—C6—C7—C8	−0.6 (3)	O6—C17—C21—F5	47.2 (2)
C6—C7—C8—C9	0.0 (3)	N4—C17—C21—F5	170.82 (16)
C6—C7—C8—N3	177.81 (18)	C20—C17—C21—F5	−73.0 (2)
O4—N3—C8—C9	170.99 (19)	N5—C19—C22—C27	−141.55 (19)
O5—N3—C8—C9	−9.6 (3)	C20—C19—C22—C27	99.1 (2)
O4—N3—C8—C7	−6.9 (3)	N5—C19—C22—C23	42.6 (2)
O5—N3—C8—C7	172.6 (2)	C20—C19—C22—C23	−76.8 (2)
C7—C8—C9—C10	0.4 (3)	C27—C22—C23—C24	1.4 (3)
N3—C8—C9—C10	−177.31 (18)	C19—C22—C23—C24	177.32 (18)
C6—C5—C10—C9	−0.2 (3)	C22—C23—C24—C25	−0.4 (3)
C3—C5—C10—C9	−176.53 (18)	C23—C24—C25—C26	−1.1 (3)
C8—C9—C10—C5	−0.3 (3)	C23—C24—C25—N6	176.76 (18)
O1—C1—C11—F2	−72.7 (2)	O10—N6—C25—C24	170.3 (2)
N1—C1—C11—F2	50.2 (2)	O9—N6—C25—C24	−10.7 (3)
C4—C1—C11—F2	167.44 (15)	O10—N6—C25—C26	−11.8 (3)
O1—C1—C11—F3	166.91 (15)	O9—N6—C25—C26	167.2 (2)
N1—C1—C11—F3	−70.3 (2)	C24—C25—C26—C27	1.4 (3)
C4—C1—C11—F3	47.0 (2)	N6—C25—C26—C27	−176.41 (19)
O1—C1—C11—F1	47.7 (2)	C25—C26—C27—C22	−0.3 (3)
N1—C1—C11—F1	170.49 (16)	C23—C22—C27—C26	−1.1 (3)
C4—C1—C11—F1	−72.3 (2)	C19—C22—C27—C26	−176.98 (19)
C3—C4—C12—O3	−62.2 (2)	C19—C20—C28—O8	−58.2 (2)
C1—C4—C12—O3	60.6 (3)	C17—C20—C28—O8	64.0 (2)
C3—C4—C12—C13	118.2 (2)	C19—C20—C28—C29	121.5 (2)
C1—C4—C12—C13	−118.9 (2)	C17—C20—C28—C29	−116.3 (2)
O3—C12—C13—C14	167.7 (2)	O8—C28—C29—C30	168.8 (2)
C4—C12—C13—C14	−12.8 (3)	C20—C28—C29—C30	−10.9 (3)
O3—C12—C13—S1	−9.5 (3)	O8—C28—C29—S2	−10.4 (3)
C4—C12—C13—S1	170.01 (15)	C20—C28—C29—S2	169.89 (14)
C16—S1—C13—C14	1.81 (19)	C32—S2—C29—C30	1.98 (19)
C16—S1—C13—C12	179.52 (18)	C32—S2—C29—C28	−178.64 (19)
C12—C13—C14—C15	−179.3 (2)	C28—C29—C30—C31	179.5 (2)
S1—C13—C14—C15	−1.9 (2)	S2—C29—C30—C31	−1.3 (2)
C13—C14—C15—C16	1.1 (3)	C29—C30—C31—C32	−0.4 (3)
C14—C15—C16—S1	0.2 (3)	C30—C31—C32—S2	1.9 (3)
C13—S1—C16—C15	−1.2 (2)	C29—S2—C32—C31	−2.3 (2)

### Hydrogen-bond geometry (Å, °)

**Table:**

*D*—H···*A*	*D*—H	H···*A*	*D*···*A*	*D*—H···*A*
O12—H12A···O6	0.85 (3)	2.28 (3)	2.867 (2)	126 (2)
O12—H12A···O8	0.85 (3)	2.20 (3)	2.949 (2)	147 (3)
O12—H12B···O7^i^	0.79 (3)	2.11 (3)	2.852 (2)	156 (3)
N1—H1···O12^ii^	0.99 (2)	2.00 (2)	2.969 (2)	168.5 (19)
N2—H2···O12^iii^	0.80 (3)	2.15 (3)	2.905 (2)	159 (2)
N5—H5···O11	0.82 (3)	2.15 (3)	2.903 (2)	153 (3)
N4—H4···O11^iv^	0.84 (3)	2.17 (3)	2.991 (2)	166 (2)
O6—H6···O2^v^	0.82 (3)	1.86 (3)	2.684 (2)	179 (3)
O1—H1A···O7^vi^	0.93 (4)	1.76 (4)	2.684 (2)	175 (4)
O11—H11A···O2^vii^	0.81 (3)	2.06 (3)	2.849 (2)	166 (3)
O11—H11B···O3^viii^	0.83 (3)	2.23 (3)	2.968 (2)	148 (3)
O11—H11B···O1^viii^	0.83 (3)	2.25 (3)	2.863 (2)	131 (3)

Symmetry codes: (i) *x*, *y*+1, *z*; (ii) −*x*+1, −*y*+1, *z*−1/2; (iii) −*x*+3/2, *y*−1/2, *z*−1/2; (iv) *x*−1/2, −*y*+1/2, *z*; (v) −*x*+1, −*y*+1, *z*+1/2; (vi) −*x*+1, −*y*, *z*−1/2; (vii) −*x*+3/2, *y*−1/2, *z*+1/2; (viii) −*x*+3/2, *y*+1/2, *z*+1/2.
